# Wip1 inhibitor GSK2830371 inhibits neuroblastoma growth by inducing Chk2/p53-mediated apoptosis

**DOI:** 10.1038/srep38011

**Published:** 2016-12-19

**Authors:** Zhenghu Chen, Long Wang, Dayong Yao, Tianshu Yang, Wen-Ming Cao, Jun Dou, Jonathan C. Pang, Shan Guan, Huiyuan Zhang, Yang Yu, Yanling Zhao, Yongfeng Wang, Xin Xu, Yan Shi, Roma Patel, Hong Zhang, Sanjeev A. Vasudevan, Shangfeng Liu, Jianhua Yang, Jed G. Nuchtern

**Affiliations:** 1 Department of Ophthalmology, Shanghai Tenth People’s Hospital, Tongji University School of Medicine, Shanghai 200072, P. R. China; 2 Texas Children’s Cancer Center, Department of Pediatrics, Dan L. Duncan Cancer Center, Baylor College of Medicine, Houston, Texas 77030, USA; 3 Department of Pathology, University of Texas MD Anderson Cancer Center, Houston, Texas 77030, USA; 4 Department of Acupuncture, First Affiliated Hospital, Heilongjiang University of Chinese Medicine, Harbin, Heilongjiang 150040, China; 5 Department of Urology, First Affiliated Hospital, Harbin Medical University, Harbin, Heilongjiang 150001, China; 6 Department of Medical Oncology, Zhejiang Cancer Hospital, Hangzhou, Zhejiang 310022, China; 7 Xinjiang Key Laboratory of Plant Resources and Natural Products Chemistry, Xinjiang Technical Institute of Physics and Chemistry, Chinese Academy of Sciences, Urumqi, Xinjiang 830011, China; 8 Division of Pediatric Surgery, Michael E. DeBakey Department of Pediatric Surgery, Dan L. Duncan Cancer Center, Baylor College of Medicine, Houston, Texas 77030, USA; 9 Department of Stomatology, Huashan Hospital, Fudan University, Shanghai 200040, China

## Abstract

Neuroblastoma (NB) is the most common extracranial tumor in children. Unlike in most adult tumors, tumor suppressor protein 53 (p53) mutations occur with a relatively low frequency in NB and the downstream function of p53 is intact in NB cell lines. Wip1 is a negative regulator of p53 and hindrance of Wip1 activity by novel inhibitor GSK2830371 is a potential strategy to activate p53’s tumor suppressing function in NB. Yet, the *in vivo* efficacy and the possible mechanisms of GSK2830371 in NB have not yet been elucidated. Here we report that novel Wip1 inhibitor GSK2830371 induced Chk2/p53-mediated apoptosis in NB cells in a p53-dependent manner. In addition, GSK2830371 suppressed the colony-formation potential of *p53* wild-type NB cell lines. Furthermore, GSK2830371 enhanced doxorubicin- (Dox) and etoposide- (VP-16) induced cytotoxicity in a subset of NB cell lines, including the chemoresistant LA-N-6 cell line. More importantly, GSK2830371 significantly inhibited tumor growth in an orthotopic xenograft NB mouse model by inducing Chk2/p53-mediated apoptosis *in vivo*. Taken together, this study suggests that GSK2830371 induces Chk2/p53-mediated apoptosis both *in vitro* and *in vivo* in a p53 dependent manner.

Neuroblastoma (NB) is widely known as a pediatric tumor that arises from the precursor cells of the sympathetic nervous system. It is the most common extracranial solid tumor in children, accounting for 8–10% of all childhood cancers and 15% of all pediatric cancer mortality^1^. Although understanding of the cancer biology of NB has improved significantly over the past three decades, this has not led to a qualitative improvement in the overall survival of high-risk NB patients. Recent advances in genomics have not revealed potentially targetable somatic mutations in high-risk NB tumors, thus there are few novel approaches with the potential to improve outcomes^2^.

p53 is one of the most important regulators in a variety of signaling pathways, and is a potent tumor suppressor^3^. It accumulates and binds to DNA upon cellular stress and activates a number of transcriptional targets including p21, PUMA, and BAX, leading to cell cycle arrest, senescence and/or apoptosis^4^. Given its anti-tumor function, p53 is mutated in more than 50% of human cancers, abrogating cell cycle arrest and apoptotic signaling responses to DNA damage and oncogenic stress^5^. However, unlike in most adult tumors, p53 mutations occur with a relatively low frequency in primary NB tumors, and p53 downstream signaling pathways remain functional, ready to induce apoptosis upon activation^2^,^6^,^7^. Cytoplasmic sequestration is an alternative molecular mechanism of p53 inactivation in NB^8^,^9^,^10^,^11^. Therefore, pharmacological reactivation of p53 by small molecules is a new strategy that is becoming an area of increasing interest in NB therapy^12^. In addition, mouse double minute 2 homolog (MDM2) is one of the transcriptional targets of p53 and destabilizes p53 by promoting its proteasome-mediated degradation via E3 ubiquitin ligase activity^13^. Several small molecules, including the MDM2 antagonist Nutlin-3a and the USP7 inhibitor P22077, have been reported to suppress tumor growth in a chemoresistant NB model by activating the p53 pathway^14^,^15^. Although the inhibitory mechanisms of these small molecules on NB are different, it is reasonable that combinatory therapy with these inhibitors may achieve better outcome for NB patients.

The type 2C family of protein phosphatases (PP2C) consists of over seven isoforms, each of which is involved in the cellular stress response^16^. Among the PP2C family members, Wip1 (wild-type *p53*-inducible phosphatase 1) stands out for its unique characteristics. Wip1 is encoded by the oncogene *PPM1D*, and its expression is induced by DNA damage in a p53-dependent manner^17^. Previous studies have shown that direct phosphorylation of p53 at serine 15 and checkpoint kinase 2 (Chk2) at threonine 68 in response to DNA damage have been reported to be responsible for Chk2/p53-mediated apoptosis^18^,^19^. Wip1 can negatively regulate the DNA damage response and enhance chemoresistance in tumor cells by dephosphorylating key proteins, including p53 (S15), Chk2 (T68), p38, ataxia telangiectasia mutated (ATM), and MDM2^20^. Therefore, inhibition of Wip1 phosphatase activity may reactivate Chk2/p53 and induce Chk2/p53-mediated apoptosis. Moreover, amplification of the *PPM1D* gene locus on 17q23 has been frequently reported in various cancers, including primary NB tumors^21^. Previous studies have shown that high expression of PPM1D predicts poor outcome in NB patients, which suggests *PPM1D* may play a critical role in the tumorigenesis of NB^22^ and therefore may have value as a therapeutic target in NB. Yet, the *in vivo* efficacy of Wip1 inhibitors is still poorly understood.

Wip1 has recently been reported to be a therapeutic target for NB therapy through gene expression analysis and *in vitro* phosphatase assays^22^. GSK2830371, a novel Wip1 inhibitor, is a selective, allosteric inhibitor of Wip1 phosphatase that binds to a unique flap subdomain of the enzyme^23^. However, the anti-tumor effect of GSK2830371 *in vivo* and the possible mechanisms in NB remained unknown. Here, we report that GSK2830371 exhibits potent cytotoxicity in *p53* wild-type NB cell lines by inducing Chk2/p53-mediated apoptosis. GSK2830371 also augments chemotherapeutic efficacy and sensitizes the chemo-resistant NB cell line LA-N-6 to traditional chemotherapeutic drugs like doxorubicin (Dox) and etoposide (VP-16). More importantly, GSK2830371 revealed anti-tumor efficacy in an orthotopic xenograft NB mouse model by inducing Chk2/p53-mediated apoptosis *in vivo*. Taken together, our results show that GSK2830371 exhibits *in vivo* anti-tumor efficacy in NB and that GSK2830371 alone or in combination with traditional therapeutic agents may be viable treatment strategies.

## Results

### Wip1 inhibitor GSK2830371 suppresses cell proliferation in a subset of NB cell lines

**Figure 1:**
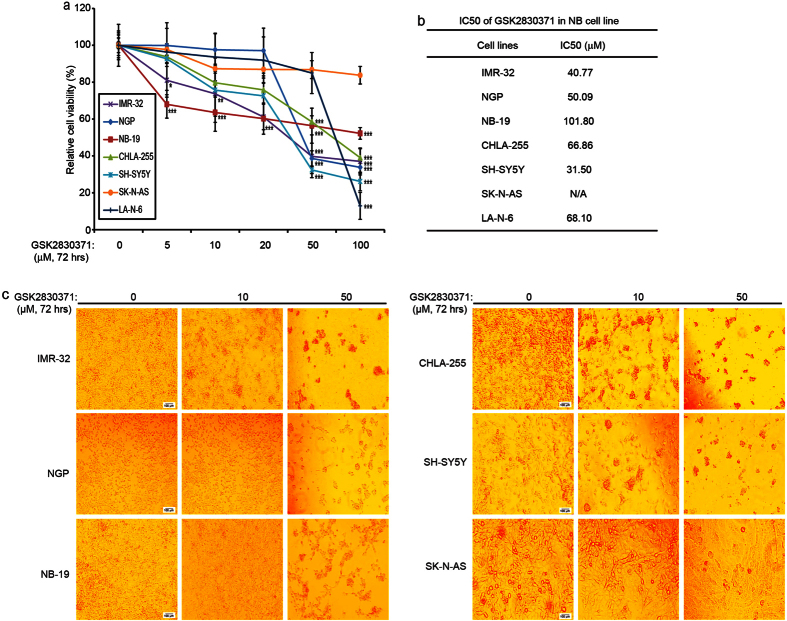
GSK2830371 shows cytotoxic effect on NB cell lines. (**a**) Seven NB cell lines including the established chemoresistant NB cell line LA-N-6, were treated with the indicated concentrations of GSK2830371 for 72 hrs. Cell viability was then assessed by a CCK-8 assay. Each experiment was repeated for six times and data were represented as % vehicle ± S.D. *P* < 0.05 (*), *P* < 0.01 (**), or *P* < 0.001 (***) (Student’s t-test, two-tailed) were indicated. (**b**) The IC50 values of GSK2830371 on each NB cell line were listed. (**c**) Morphologic changes of five *p53* wild-type NB cell lines (IMR-32, NGP, NB-19, CHLA-255 and SH-SY5Y) and one *p53* mutant NB cell line SK-N-AS with the treatment of GSK2830371 for 72 hrs were shown.

To determine the antitumor effect of GSK2830371 in NB, seven NB cell lines (IMR-32, NGP, NB-19, CHLA-255, SH-SY5Y, SK-N-AS and LA-N-6) were included in the cell viability assay. Of those NB cell lines, SK-N-AS cells are unique in that they harbor mutant *p53* and the downstream signaling of p53 is not intact in this cell line. Therefore, SK-N-AS cells do not respond to p53 activators like P22077^15^. As shown in Fig. 1a, GSK2830371 reduced the viability of all the cell lines tested except the *p53* mutant SK-N-AS cell line. The IC50 of GSK2830371 in all seven cells lines were listed in Fig. 1b.

The cytotoxic effect of GSK2830371 on NB cells except SK-N-AS cell line was further illustrated by cell morphology imaging (Fig. 1c). Consistent with this observation, flow cytometric analysis showed that GSK2830371-induced cell death in IMR-32, NGP, NB-19, CHLA-255, and SH-SY5Y but not in SK-N-AS (Supplementary Fig. S1). Our results demonstrate that GSK2830371 suppresses proliferation and induces cell death in *p53* wild-type NB cell lines.

### GSK2830371 inhibits colony formation ability of *p53* wild-type NB cells

**Figure 2:**
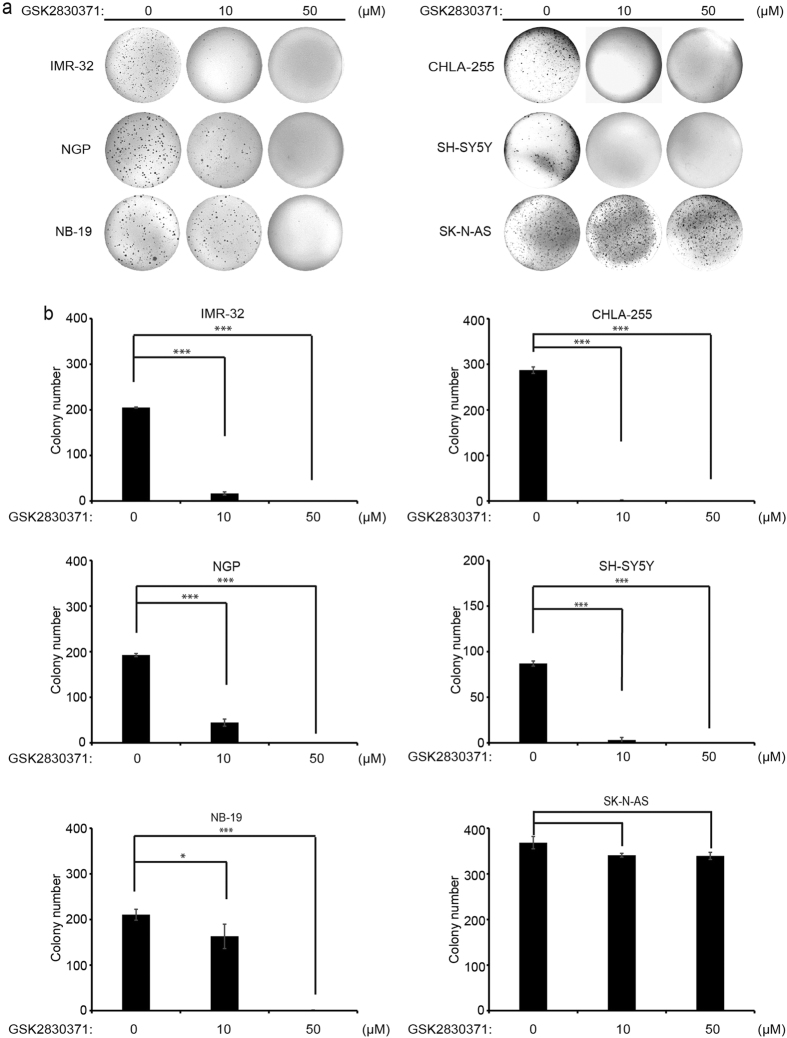
GSK2830371 suppresses the anchorage-independent growth of NB cells. (**a**) A panel of six NB cell lines were seeded in six-well plates with indicated concentrations of GSK2830371 and agar, and grown for two to three weeks. Cells were stained with crystal violet for 4 hrs, and photographed. (**b**) Colonies were counted and colony numbers were presented as % vehicle ± S.D. *P* < 0.05 (*), or *P* < 0.001 (***) (Student’s t-test, two-tailed) were indicated. Each experiment was repeated at least twice and representative results were presented.

To evaluate whether GSK2830371 inhibits the colony formation ability of NB cell lines, soft agar assays were performed using a panel of six NB cell lines. In this assay, we found that all cell lines tested except SK-N-AS showed a significant decrease in the ability to form colonies after GSK2830371 treatment, compared with the control group (Fig. 2a). Colony numbers were counted in each group, which showed that GSK2830371 significantly attenuated anchorage-independent growth in *p53* wild-type NB cell lines in a dose dependent manner (Fig. 2b).

### GSK2830371 induces Chk2/p53-mediated apoptosis in NB cells in a p53-dependent manner

**Figure 3:**
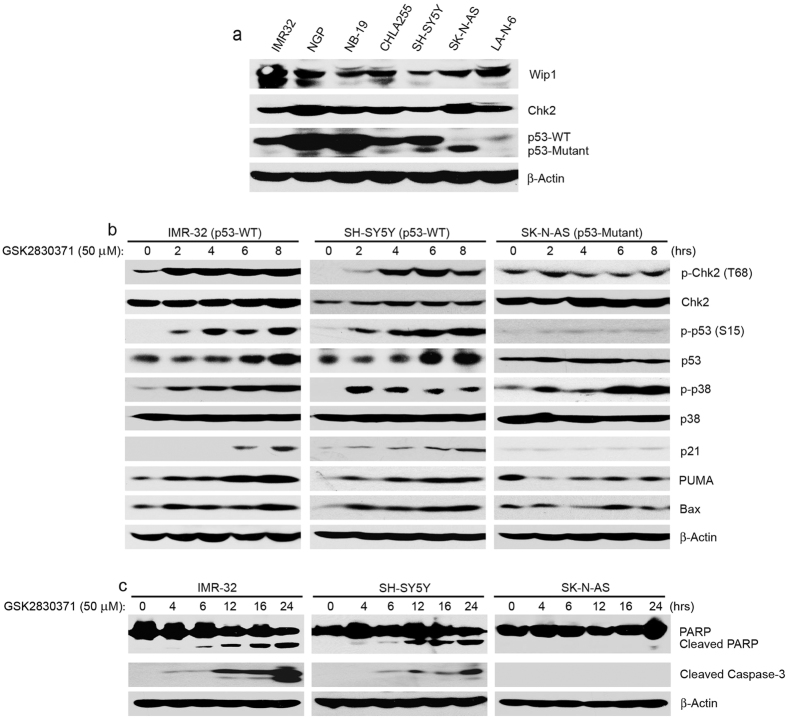
GSK2830371 increases p53 activity and induces Chk2/p53-mediated apoptosis in *p53* wild-type NB cell lines, but not in the *p53* mutant SK-N-AS cell line. (**a**) The basal expressions of Wip1, Chk2 and p53 in seven NB cell lines were detected by protein immunoblotting. (**b**) IMR-32, SH-SY5Y and SK-N-AS cells were treated with 50 μM of GSK2830371 for various time points (0–8 hrs), then the cell lysates were subjected to SDS-PAGE, and immunoblotted with the indicated antibodies. (**c**) IMR-32, SH-SY5Y and SK-N-AS cells were exposed to 50 μM of GSK2830371 for varying durations (0–24 hrs), and the cells were harvested for the protein immunoblotting assay. PARP and Caspase-3 cleavages were detected by immunoblotting with the antibodies. Each assay was repeated in triplicates and β-Actin was used as a loading control for whole cell extracts in all samples.

To understand the molecular mechanism that determines the sensitivity of NB cells to GSK2830371, we first examined the expression level of Wip1, Chk2, and p53 in the aforementioned six NB cell lines as well as in LA-N-6, a known chemo-resistant NB cell line. All the tested NB cells showed high expression of Wip1 (Fig. 3a). GSK2830371-induced apoptosis in IMR-32 and SH-SY5Y cells in a time- and dose- dependent manner, but not in SK-N-AS cells (Fig. 3c and Supplementary Fig. S2).

Wip1 is known to negatively regulate a number of response genes upon DNA damage by dephosphorylating Chk2 (T68), p53 (S15), and p38 (Thr180/Tyr182). Also, inhibition of Wip1 by Arsenic Trioxide enhances Chk2/p53-mediated apoptosis^24^. To determine the mechanisms that are responsible for GSK2830371-induced apoptosis in NB cells, we checked the expression levels of Chk2 (T68), p53 (S15), and p38 (Thr180/Tyr182) in IMR-32, SH-SY5Y and SK-N-AS cells after GSK2830371 treatment. Richter *et al*. reported that GSK2830371 increased the presence of p-p53 (S15), p53, p21, and PUMA protein levels in IMR-32 cells, but not in SK-N-AS cells, whereas the phosphorylation of Chk2 (T68) increased in both IMR-32 and SK-N-AS cells^22^. Here we found that GSK2830371 increased the phosphorylation of Chk2 (T68) and p53 (S15) in *p53* wild-type NB cells (IMR-32 and SH-SY5Y). However, this effect was not pronounced in the *p53* mutant SK-N-AS cells (Fig. 3b). As expected, the p53 protein levels were stabilized by GSK2830371 in both IMR-32 and SH-SY5Y cells, but not in SK-N-AS cells. Consistently, treatment with GSK2830371 resulted in increased expressions of p21, PUMA, and Bax in IMR-32 and SH-SY5Y cells, but not in SK-N-AS cells (Fig. 3b).

Interestingly, an increase of phosphorylated p38 (Thr180/Tyr182) was also observed upon GSK2830371 treatment in all of the cell lines tested (Fig. 3b). p38 has also been reported to play an essential role in cell apoptosis pathways^25^,^26^,^27^ and inactivation of Wip1 is known to upregulate p38 MAPK phosphatase activity and suppress tumor growth *in vivo*^28^. We then examined whether p38 inhibition affects GSK2830371-induced cell death in NB cells. We tested the effect of the p38 inhibitor SB203580 on GSK2830371 function in SH-SY5Y cells. Compared with GSK2830371 treatment alone, the combination therapy of SB203580 and GSK2830371 led to decreased levels of PARP cleavage, p53 and its downstream apoptotic effector PUMA and Bax after 6 hrs of treatment, indicating that p38 activation by GSK2830371 potentiates p53 activation and its tumor suppressive function in NB cells (Supplementary Fig. S3).

To further elucidate the role of p53 in the mechanism of action of GSK2830371 in NB cells, we used the CRISPR/Cas9-mediated genomic editing technology to generate a *p53*-knock out (KO) SH-SY5Y cell line. Cell viability assay revealed that *p53*-KO SH-SY5Y cells were more resistant to GSK2830371-mediated cytotoxic effect compared with the vehicle control SH-SY5Y cells (Supplementary Fig. S4a). Consistent with these results, GSK2830371 was not effective in inducing cell death in *p53*-KO SH-SY5Y cells (Supplemental Fig. S4b). However, GSK2830371-induced p38 activation was still intact in *p53*-KO SH-SY5Y cells. GSK2830371 treatment induced PARP cleavage and increased the protein levels of p-p53 (S15) and p53, as well as p53 downstream effectors p21, PUMA, and Bax, in the vehicle control SH-SY5Y cells, and importantly, the induction of these genes by GSK2830371 was abolished in the *p53*-KO SH-SY5Y cell line. These results show that the cytotoxic effects of GSK2830371 were dependent on p53. Taken together, these data suggest that GSK2830371-induced p53 activation plays an essential role in GSK2830371-induced cytotoxicity and p38 acts as the upstream of p53 and functions through p53 in NB cells.

### GSK2830371 enhances the cytotoxic effect of Dox and VP-16 in *p53* wild-type NB cell lines

**Figure 4:**
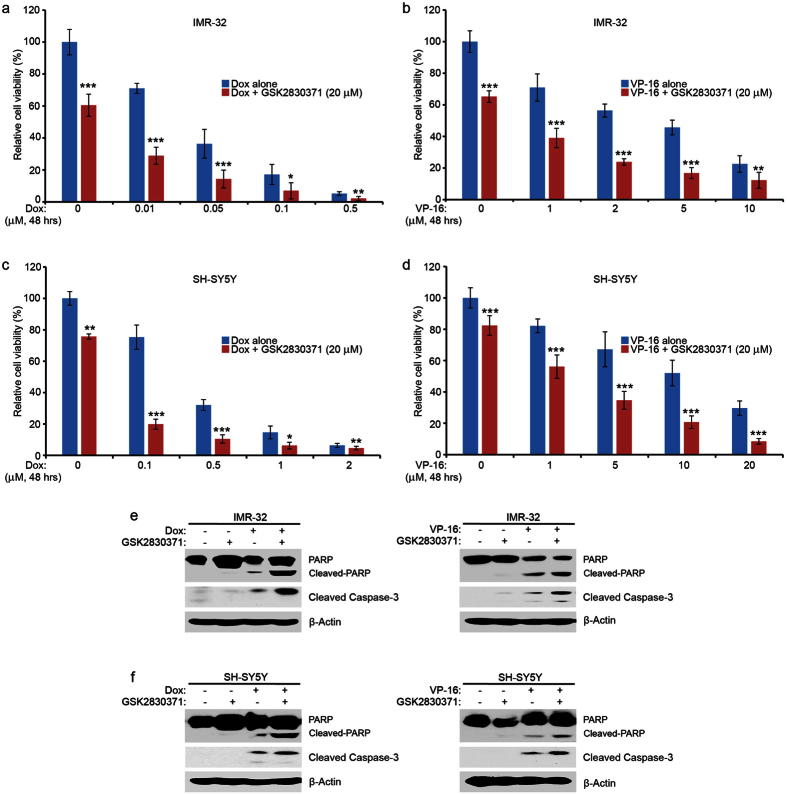
GSK2830371 enhances Dox- and VP-16-induced cytotoxicity in *p53* wild-type NB cell lines. (**a** and **c**) IMR-32 and SH-SY5Y cells were seeded in 96-well plates and were incubated with the indicated concentrations of Dox plus DMSO or 20 μM of GSK2830371 for 48 hrs. Cell viability was then measured by the CCK-8 assay as described in Methods. (**b** and **d**) IMR-32 and SH-SY5Y cells were seeded in 96-well plates and were incubated with the indicated concentrations of VP-16 plus DMSO or 20 μM of GSK2830371 for 48 hrs. The cell viability was then measured by the CCK-8 assay. The results were represented as % vehicle ± S.D. *P* < 0.05 (*), *P* < 0.01 (**) or *P* < 0.001 (***) (Student’s t-test, two-tailed) were indicated. (**e**) IMR-32 cells were treated with Dox (0.5 μM), or VP-16 (5 μM), or GSK2830371 (20 μM) alone, or their combinations for 8 hrs. (**f**) SH-SY5Y cells were treated with Dox (1 μM) alone, or VP-16 (5 μM), or GSK2830371 (20 μM) alone, or their combinations for 8 hrs. At the end of the treatment, cells were collected and then the lysates were subjected to SDS-PAGE, and immunoblotted with the indicated antibodies. β-Actin was used as a loading control for whole cell extracts in all samples. Each experiment was repeated for at least three times.

Loss of Wip1 function has been reported to sensitize mouse embryonic fibroblasts to stress-induced apoptosis via a p38/p53 induced mechanism^29^, therefore, it’s reasonable that pharmacological inhibition of Wip1 would potentiate p53 activity and result in increased chemo-sensitivity in NB cell lines. To test this idea, IMR-32 and SH-SY5Y cells were treated with Dox or VP-16 in combination with or without GSK2830371. Previous studies suggest that GSK2830371 has synergistic anti-proliferative activity with Dox in IMR-32 cells^22^. Several other studies reported that GSK2830371 sensitized tumor cells to a genotoxic response in combination with MDM2 antagonist nutlin-3a in a p53 dependent manner^30^,^31^,^32^,^33^. In our study, as shown in Fig. 4a–d, GSK2830371 enhanced the cytotoxic effect of Dox or VP-16 on both IMR-32 and SH-SY5Y cell lines. As expected, GSK2830371 also augmented Dox- and VP-16-induced apoptosis in IMR-32 and SH-SY5Y cells by enhancing the induction of PARP and Caspase-3 cleavages (Fig. 4e,f). In contrast, GSK2830371 did not sensitize the *p53* mutant cell line SK-N-AS to Dox or VP-16 treatment (Supplementary Fig. S5). These results demonstrate that GSK2830371 enhances Dox- and VP-16- induced cytotoxicity in *p53* wild-type NB cell lines in a p53-dependent manner.

### GSK2830371 sensitizes the chemoresistant LA-N-6 cells to Dox and VP-16 treatment

**Figure 5:**
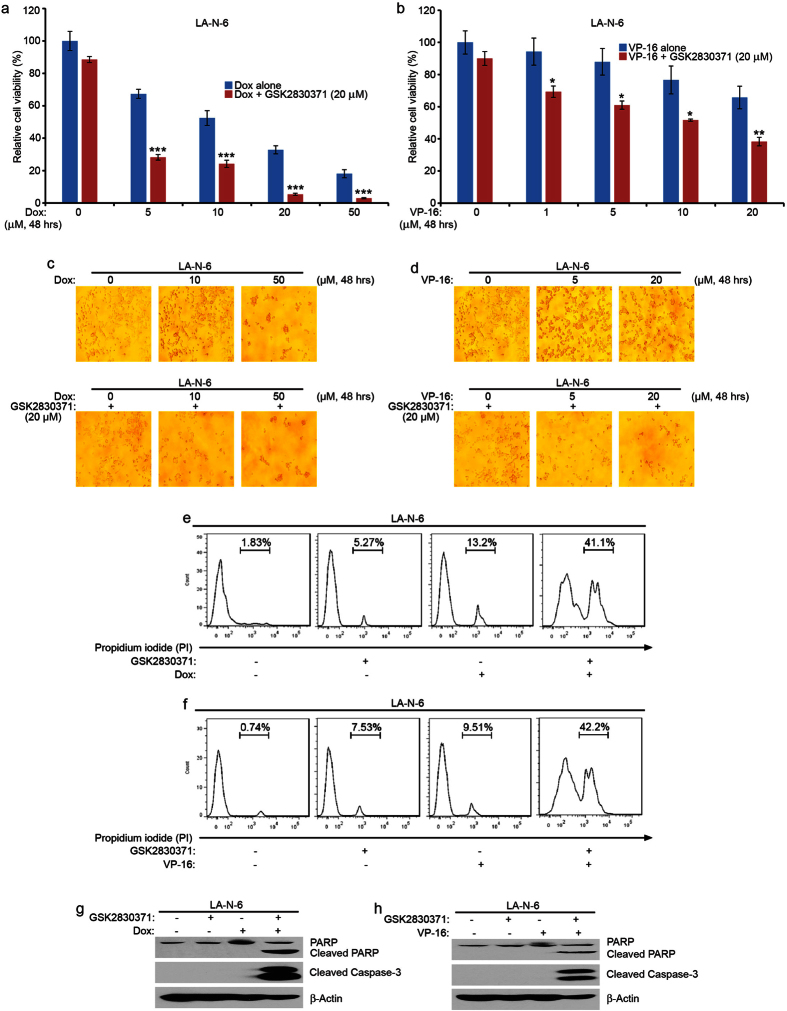
GSK2830371 enhances Dox- and VP-16-induced cytotoxicity in the chemoresistant LA-N-6 cell line. LA-N-6 cells were seeded in 96-well plates and the cells were incubated with Dox (**a**) or VP-16 (**b**) at the indicated concentrations with or without 20 μM of GSK2830371 for 48 hrs. The cell viability was then measured using the CCK-8 assay. Results are presented as % vehicle ± S.D. *P* < 0.05 (*), *P* < 0.01 (**) or *P* < 0.001 (***) (Student’s t-test, two-tailed) were indicated. (**c** and **d**) LA-N-6 cells were treated with Dox or VP-16 alone or with GSK2830371 (20 μM) at the indicated concentrations and cell morphology was captured using an optical microscope. (**e** and **f**) LA-N-6 cells were treated with Dox (1 μM) alone, or VP-16 (5 μM) alone, or GSK2830371 (20 μM) alone, or their combinations for 36 hrs. At the end of the treatment, the cells were fixed and examined by flow cytometry using PI staining to label apoptotic cells. (**g** and **h**) LA-N-6 cells were treated with Dox alone (1 μM), or VP-16 (5 μM) alone, or GSK2830371 (20 μM) alone, or their combinations for 36 hrs. The cells were harvested and subjected to SDS-PAGE, and immunoblotted with the indicated antibodies. β-Actin was used as a loading control for whole cell extracts in all samples and each assay was repeated in triplicates.

To test whether GSK2830371 could overcome established chemoresistance in NB cells, the chemoresistant LA-N-6 cell line was treated with varying doses of Dox and VP-16, with or without GSK2830371. Demonstrated in Fig. 5a–d, GSK2830371 augmented Dox- and VP-16-induced cytotoxicity on LA-N-6 cells. These results were further confirmed by cell morphology changes and propidium iodide (PI) staining (Fig. 5e,f). As shown in Fig. 5g,h, GSK2830371 significantly enhanced Dox- or VP-16-induced apoptosis in LA-N-6 cells. These data indicate that GSK2830371 could overcome established chemoresistance in LA-N-6 cells by enhancing Dox- and VP-16-induced cytotoxicity.

### GSK2830371 significantly inhibits NB tumor growth and induces Chk2/p53-mediated apoptosis in an orthotopic xenograft NB mouse model

An orthotopic xenograft NB mouse model was utilized to test whether Wip1 inhibition by GSK2830371 could inhibit NB tumor growth *in vivo*. Luciferase-transduced SH-SY5Y cells were surgically injected into the left renal capsule of nude mice. Two weeks after injection, tumor signals were detected by bioluminescent imaging. Tumor bearing mice were randomly divided into two groups and treated with either dimethyl sulfoxide (DMSO) (carrier control) or GSK2830371, which was administered alone by intraperitoneal injection at 25 mg/kg daily for 21 days.

**Figure 6:**
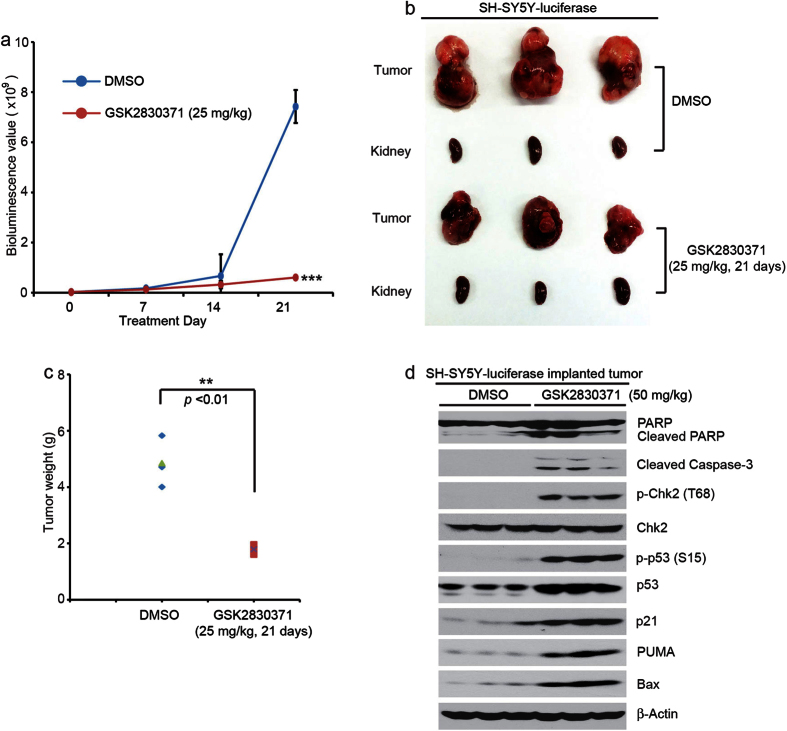
GSK2830371 inhibits tumor growth in an orthotopic xenograft NB mouse model. (**a**) The bioluminescence values of the DMSO control group and GSK2830371 treated group (25 mg/kg) were measured every week upon treatment. *P* < 0.001 (***) (Student’s t-test, two-tailed) was indicated. (**b**) Photographs of SH-SY5Y xenografted tumors and the corresponding kidney controls from DMSO control group and GSK2830371 treated group (25 mg/kg) were taken at the end of treatment (21 days). (**c**) SH-SY5Y-derived tumor weights from control (N = 3) and treatment groups (N = 3) are presented as the mean with SDs. *P* < 0.01 (**) (Student’s t-test, two-tailed) was indicated. (**d**) The mice bearing SH-SY5Y xenografted tumors for 4 weeks were treated with 50 mg/kg of GSK2830371 by intraperitoneal injection once. Four hours later, the mice were sacrificed and the tumors were harvested and lysed for immunoblotting with the indicated antibodies. β-Actin was detected as a loading control.

For the implanted SH-SY5Y cells bearing luciferase activity, we obtained bioluminescence values every week to evaluate tumor size over time. After 14 days, there was a rapid growth of the tumors in the control group while the tumors of GSK2830371 treated group grew much more slowly (Fig. 6a). Treatment with GSK2830371 significantly inhibited tumor growth compared with the control (Fig. 6b,c). In addition, there were no obvious signs of drug related toxicity as measured by gross mouse weight during the study (Supplementary Fig. S6).

Tumor bearing mice treated with GSK2830371 at 50 mg/kg once, and the tumors were harvested 4 hrs later. The tumor tissues were then lysed and analyzed by protein immunoblotting assay. Consistent with the *in vitro* data, GSK2830371 significantly induced tumor cell apoptosis by increasing the phosphorylation levels of Chk2 (T68), p53 (S15), as well as protein levels of p53, p21, PUMA, and Bax (Fig. 6d). These results indicate that GSK2830371 inhibits NB tumor growth and induces Chk2/p53-mediated apoptosis *in vivo.*

## Discussion

Restoring p53 function is a potential strategy in NB therapy, and the screening of small molecules that can reactivate p53 is an active area in cancer research^34^,^35^. Here we report that GSK2830371, a novel Wip1 inhibitor, is a potent p53 activator in NB. GSK2830371 inhibits the proliferation and colony formation ability in a subset of NB cells. It also induces Chk2/p53-mediated apoptosis in NB cells. Furthermore, GSK2830371 sensitizes *p53* wild-type NB cell lines, including the known chemoresistant LA-N-6 cells, to the treatment of traditional chemotherapeutic agents like Dox and VP-16. More importantly, GSK2830371 significantly inhibited NB tumor growth in an orthotopic xenograft NB mouse model by inducing Chk2/p53-mediated apoptosis.

Tumor suppressor p53 is an important tumor suppressor that plays an essential role in maintaining genomic integrity. The activation of the checkpoint kinase Chk2 and p53 upon cellular stress could result in Chk2/p53-mediated apoptosis^24^. We found that the cytotoxic effect of GSK2830371 on NB cell lines is p53-dependent, as GSK2830371 was not effective on the *p53* mutant SK-N-AS cell line. In addition, p38 activation by GSK2830371 may also play a role in GSK2830371-induced p53 activation and apoptosis in NB cells. To determine the mechanisms that are responsible for GSK2830371-induced cytotoxicity in NB cells, a *p53*-knock out (KO) SH-SY5Y cell line was generated by using the CRISPR/Cas9-mediated genomic editing technology. Compared with the vehicle control SH-SY5Y cells, GSK2830371 was not effective in inhibiting cell proliferation and inducing cell death on the *p53*-KO SH-SY5Y cells (Supplementary Fig. S4). However, GSK2830371-induced p38 activation was still intact in *p53*-KO SH-SY5Y cells. These data suggest that GSK2830371-induced p53 activation plays an essential role in GSK2830371-induced cytotoxicity and p38 acts as the upstream of p53 and functions through p53 in NB cells.

Wip1 encoding gene *PPM1D* has been found to be amplified in primary NB tumors by way of chromosome 17q23 gain^36^. NB patients with high Wip1 expression also had significantly shorter overall survival rate than those with low expression of *PPM1D*/Wip1 (R2: http://r2.amc.nl), which indicates that Wip1 may be a potential target in NB therapy, as Richter *et al*. reported before^22^. Previous studies have shown that chemical inhibition of Wip1 phosphatase results in the suppression of tumorigenesis^37^. Therefore, the discovery of selective and high-affinity inhibitors of Wip1 is crucial. Some of them are substrate-based cyclic phosphopeptides that act as competitors to Wip1, but these peptides suffer from poor absorption or permeation into cells because of their large molecular weight^38^. CCT007093, the first generation chemical inhibitor of Wip1, has off-targets effects^39^. 2,4-bisarylthiazoles induce apoptosis of *PPM1D* amplified cell-lines, but without significant inhibition of Wip1 phosphatase activity^40^. Using a Wip1 phosphatase-activity-based screening of a diverse library of chemical compounds, several potential candidates display anti-tumor effect in cell lines. However, serious short- and long-term toxicities in patients and mixed specificity may limit the clinical application of these compounds^41^.

Different from traditional substrate-derived competitive inhibitors, GSK2830371 acts as an antagonist of Wip1 by binding to a unique structural ‘flap’ subdomain of Wip1 and allosterically inhibits Wip1 phosphatase activity in a domain guided and dependent manner^23^. Wip1 structurally differs from other PP2C family members as its ‘flap’ subdomain is located near the catalytic site, therefore the ‘flap’ subdomain-dependent small molecule GSK2830371 demonstrates much higher selectivity and efficacy for Wip1 over other phosphatases. In our study, GSK2830371 selectively induced cell death in *p53* wild-type NB cell lines but not in *p53* mutant SK-N-AS cell line, which confirms its selective activation of p53 in NB cells. To determine the IC50s of GSK2830371 in NB cells, we seeded 1 × 10^4^ IMR-32 and SH-SY5Y cells in 96 well plates and treated them with GSK2830371 for 3 days (72 hrs), while Richter *et al*. seeded 500 cells per well in 96-well plates and treated the cells with GSK2830371 for up to 7 days^22^. It is likely that when cells are treated with the drug for a longer time, a much lower IC50 will be observed. Thus, our IC50 values of GSK2830371 in NB cells are higher than that reported by Richter *et al*. Moreover, GSK2830371 shows high efficiency in inducing Chk2/p53-mediated apoptosis in NB cell lines. Similar results are obtained from tumor tissues generated in an orthotopic xenograft NB mouse model. In addition, no obvious health problems or weight loss was observed in the treatment groups compared to the control group, indicating the low toxicity of GSK2830371 *in vivo*.

Chemoresistance arises frequently as one of the major causes in patients with relapse, especially in high-risk NB patients^42^. Inhibition of C-terminal truncated Wip1 is reported to enhance the effect of Dox in human colorectal carcinoma cell lines^43^. Here we found that GSK2830371 enhanced Dox- and VP-16-induced cytotoxicity in IMR-32 and SH-SY5Y cells. Furthermore, GSK2830371 also sensitized the chemo-resistant NB cell line LA-N-6 to Dox and VP-16 treatment. These results suggest that the combination of Wip1 inhibitor GSK2830371 with traditional chemotherapeutic drugs like Dox and VP-16 might be a viable option for NB patients.

In conclusion, small molecule inhibitor GSK2830371 inhibits NB cell proliferation and suppresses NB tumor growth primarily by reactivating p53 and inducing Chk2/p53-mediated apoptosis. In addition, p38 activation by GSK2830371 also plays a role in GSK2830371-induced cytotoxicity. GSK2830371 enhances Dox- and VP-16-induced cytotoxicity and overcomes established chemoresistance in LA-N-6 cell line in combination with the traditional therapeutic agents. GSK2830371 also demonstrates *in vivo* anti-tumor efficacy in an orthotopic xenograft NB mouse model by inducing Chk2/p53-mediated apoptosis. Our study provides a basis for the rational use of Wip1 inhibitors like GSK2830371 alone or in combination with traditional therapeutic agents such as Dox and VP-16 as treatment options for NB patients.

## Materials and Methods

### Antibodies and Reagents

Wip1 inhibitor GSK2830371 was purchased from ChemieTek (CT-GSK283) (ChemieTek, Indianapolis, IN, USA). Doxorubicin (Dox, D1515), etoposide (VP-16, E1383), and anti-β-Actin (A2228) antibodies were purchased from Sigma (Sigma-Aldrich Corp, St. Louis, MO, USA). Anti-*PPM1D* (Wip1) (A300-664A) antibodies were purchased from Bethyl Laboratories (Bethyl Laboratories, Inc., Montgomery, TX, USA). Anti-p53 Antibodies (DO-1) (sc-126) and p21 Antibodies (SX118) (sc-53870) were purchased from Santa Cruz Biotechnology (Santa Cruz Biotechnology, Dallas, TX, USA). Anti-p-Chk2 (Thr68) (C13C1) (2197S), anti-Chk2 (D9C6) (6334S), anti-p-p53 (Ser15) (9284S), anti-phospho-p38 (Thr180/Tyr182) (9211S), anti-p38 (9212S), anti-PARP (9532S), anti-Caspase-3 (9662S), anti-Mouse, (7076S) and anti-Rabbit (7074S) antibodies were purchased from Cell Signaling Technology (Cell Signaling Technology, Danvers, MA, USA).

### Cell Lines and Cell Culture

The MYCN-amplified (IMR-32, NGP and NB-19), and MYCN-non-amplified (CHLA-255, SK-N-AS and SH-SY5Y) human NB cell lines were cultured in RPMI Medium 1640 (RPMI) (Lonza, Walkersville, MD, USA) supplemented with 10% (v/v) heat-inactivated Fetal Bovine Serum (FBS) (SAFC Biosciences, Lenexa, KS, USA), 100 units/mL penicillin, and 100 μg/mL streptomycin. The chemoresistant NB cell line LA-N-6 was grown in RPMI containing 20% (v/v) heat-inactivated FBS, 100 units/mL penicillin, and 100 μg/mL streptomycin. All cells were maintained at 37 °C in a humidified incubator with 5% CO_2_. All experiments were performed with cells under exponential growth conditions. The NB-19 cells came from Dr. A. Davidoff (St. Jude’s Children’s Hospital) and LA-N-6 cells were provided by Dr. R. Seeger (Children’s Hospital of Los Angeles), respectively. The SH-SY5Y cell line with stable expression of luciferase was generated by transfection with a pcDNA3 luciferase expression plasmid into the cells. A stable cell line was established after 10 days of 800 μg/ml G418 (Enzo Life Sciences, Farmingdale, NY, USA) selection.

### Cell Viability Assay

Cell viability assays were performed using the Cell Counting Kit-8 (CCK-8, WST-8[2-(2-methoxy-4-nitrophenyl)-3-(4-nitrophenyl)-5-(2,4-disulfophenyl)-2 H-tetrazolium, monosodium salt]) (Dojindo Laboratories, Rockville, MA, USA). Cells were plated and grown in 96-well clear-bottom plates starting at 1 × 10^4^ cells/well. After 24 hrs of incubation, the media were changed and increasing concentrations of GSK2830371, Dox, VP-16 or their combinations were added to the wells and the cells were then incubated at 37 °C for 48 hrs or 72 hrs. Then a mixture of 10 μL of CCK-8 and 190 μL of RPMI with 10% FBS was added into each well. After one hour of incubation, the absorbance was measured at 450 nm using a microplate reader. Each experiment was performed in replicates of six and background reading of the media was subtracted from each well to standardize the results. The IC50 values of GSK2830371 on each cell line listed was calculated by using Prism 5.0 (Graphpadm Software Inc., La Jolla, CA), based on the data collected in the cell viability assay.

### Cell Imaging

A total of seven NB cell lines were seeded in 96-well plates at appropriate concentrations. After 48 hrs or 72 hrs of treatments with indicated concentrations of GSK2830371, Dox, VP-16, or their combinations, cell morphologies were observed and captured using an optical microscope. Each result was performed in triplicate.

### Colony Formation Assay

The soft agar assay was performed as previously described^44^,^45^. Briefly, a 5% (w/v) base agar layer was made by adding agar (214220, Difco Laboratories, Detroit, MI, USA) into distilled water and then autoclavingthe mixture for 50 min before cooling in a 56 °C water bath. This solution was then mixed with RPMI with 10% FBS to a final concentration of 0.5%. To make the bottom agar layer, 2 mL of the 0.5% agar/RPMI solution were added to each well and cooled down until semi-solid. The top agar layer was made of 1.5 ml 0.3% agar and each NB cell line was counted and added to the mixture at 1 × 10^4^ cells/well along with the indicated concentrations of GSK2830371. Cells were grown at 37 °C for 2 to 3 weeks, then stained with 500 μL of 0.005% crystal violet (C3886, Sigma). Images were captured by the microscope and colonies were counted after 4 hrs. Each assay was performed in triplicate.

### Protein Immunoblotting

The experiments were performed as described previously^46^,^47^. Briefly, after each treatment, cells were washed twice with ice cold PBS and then lysed on a rotator at 4 °C for 30 min in cooled RIPA buffer (50 mM Tris-HCl at pH 7.4, 150 mM NaCl, 1 mM EDTA, 1% NP-40, 0.25% sodium deoxycholate, 1 mM phenylmethylsulfonyl fluoride, 1 mM benzamidine, 10 μg/mL leupeptin, 1 mM dithiothreitol, 50 mM sodium fluoride, 0.1 mM sodium orthovanadate, and phosphatase inhibitor cocktail 2 and 3 (p5726 and p0044, Sigma)). After centrifuging at 13,000 rpm for 15 min, the supernatants were used as cell lysates. Protein concentrations were measured using Bradford reagent (Bio-Rad Laboratories, Hercules, CA, USA) and each sample was mixed in a 3:1 ratio (v/v) with 4× loading buffer and heated at 100 °C for 6 min. Lysates were then separated by SDS-PAGE, transferred to polyvinylidence fluoride (PVDF) membranes (BioRad), blocked with 5% milk or BSA for one hr at RT (25 °C), and probed with appropriate dilutions of indicated primary antibodies overnight at 4 °C. The membranes were then incubated with anti-mouse or rabbit IgG conjugated with horseradish peroxidase at room temperature for 1hr. The ECL-Plus Western detection system (GE Health Care, Buckinghamshire, UK) was then used for chemiluminescent visualization. β-Actin was used as a loading control for whole cell extracts.

### PI staining assay

The experiment was performed as described previously^48^. Briefly, NB cell lines were seeded in 10 cm dishes and treated with increasing concentrations of GSK2830371 for 36 hrs. Cells were trypsinized, resuspended in RPMI 1640 medium, and centrifuged at 400 × g for 5 min at 4 °C. Cells were then washed with 1× cold PBS twice and resuspended at a density of 1 × 10^6^ cells/ml in 1× binding buffer (51-66121E; BD Biosciences, San Jose, CA, USA). Then 100 μl of non-fixed cell suspension was transferred into a new tube and 5 μl of 50 μg/mL PI staining solution (51-66211E; BD Biosciences) was added into the tube. The tubes were gently vortexed and incubated for 15 min at RT (25 °C) in the dark. After adding the additional 400 μl of 1× binding buffer, the samples were analyzed by flow cytometry within 1 hr. As viable cells with intact membranes resist PI staining, only the membranes of dead cells are subject to PI staining. Unstained cells were used as a negative control and untreated cells were used as a control for the treated cells.

### Generation of the *TP53* Gene Knockout SH-SY5Y Cell Line by CRISPR/Cas9-Mediated Genomic Editing Technology

The lenti-viral vector bearing *p53* gRNA (CCTGCATGGGCGGCATGAAC) and Cas9-2A-puromycin expression cassettes were transfected into 293T cells (2.5 × 10^6^ cells/dish), together with four packaging vectors (Hgpm, Tat-1b, Rev-1b, and VSVG). The viral supernatants were collected 48 hrs later. A mixture of 4 μg/ml polybrene and the viral supernatants was used to transduce SH-SY5Y cells (5 × 10^5^ cells/well seeded in six-well plates). The viral transduced SH-SY5Y cells were selected by 0.5 μg/ml puromycin. And then the *p53* knockout (KO) single cell clone was selected later and genotyped by PCR flanking the target sites, followed by cloning the PCR products and sequencing analysis. Sequence comparison of the *p53*^*Δ19*^-KO allele and the *p53*^*Δ16*^-KO allele with a *p53* wild type allele was shown as previously reported^49^.

### Antitumor Efficacy in an Orthotopic Mouse Model of NB

Five to six-week-old female athymic NCR nude mice were purchased from Taconic (Taconic, Hudson, NY, USA) and maintained under barrier conditions (pathogen-free conditions provided by plastic cages with sealed air filters). The preclinical mouse model of NB was established using orthotopic (intrarenal) implantation of the NB cells as described previously^50^,^51^,^52^. Briefly, a transverse incision was created over the left flank of the nude mouse and 1.5 × 10^6^ human luciferase-transduced SH-SY5Y cells in 0.1 ml of PBS were surgically injected into the left renal capsule and toward the superior pole of the left kidney of the nude mice.

After allowing them to engraft for 2 to 3 weeks, mice bearing tumors with similar sizes (using bioluminescent imaging to monitor tumor growth) were randomly divided into two groups: a DMSO control group and a GSK2830371 treated group (25 mg/kg by intraperitoneal (i.p.) injection once daily for 21 days). Both control and GSK2830371 treated groups contained three mice. At the end of the treatment, all mice were sacrificed. Tumors and the right kidneys (control) were harvested, weighed, photographed.

For the protein immunoblotting, the SH-SY5Y implanted NB orthotopic mice with similar sizes were randomly divided into two groups and treated with either DMSO or GSK2830371 (50 mg/kg by intraperitoneal (i.p.) injection) once. Four hours later, the mice were sacrificed and the tumors were harvested and lysed for immunoblotting. All mice were handled according to protocols approved by the Institutional Animal Care and Use Committee of the Baylor College of Medicine.

### Statistical Analysis

All values were presented as mean ± standard deviation (SD). A two-tailed Student’s t-test was used to determine the statistical significance of *in vitro* and *in vivo* assay between the control and drug treatment groups. Each assay was repeated at least twice and representative results were presented. *P* < 0.05 was considered to be statistically significant.

## Additional Information

**How to cite this article**: Chen, Z. *et al*. Wip1 inhibitor GSK2830371 inhibits neuroblastoma growth by inducing Chk2/p53-mediated apoptosis. *Sci. Rep.* **6**, 38011; doi: 10.1038/srep38011 (2016).

**Publisher's note:** Springer Nature remains neutral with regard to jurisdictional claims in published maps and institutional affiliations.

## Supplementary Material

Supplemental Information
