# Supplementary material for: Wip1 inhibitor GSK2830371 inhibits neuroblastoma growth by inducing Chk2/p53-mediated apoptosis
Source: Sci Rep. 2016 Dec 19;6:38011. doi: 10.1038/srep38011 (PMC5171816; doi:10.1038/srep38011)
Supplement: Supplemental Information [file srep38011-s1.pdf]

Supplemental information

Wip1 inhibitor GSK2830371 inhibits neuroblastoma growth by inducing Chk2/p53-mediated apoptosis

Zhenghu Chen[1,2], Long Wang[3,4], Dayong Yao[3,5], Tianshu Yang[1], Wen-Ming Cao[3,6], Jun Dou[3,7], Jonathan C. Pang[2], Shan Guan[2], Huiyuan Zhang[2], Yang Yu[2], Yanling Zhao[2], Yongfeng Wang[2], Xin Xu[2], Yan Shi[8], Roma Patel[8], Hong Zhang[3], Sanjeev A.Vasudevan[8], Shangfeng Liu[1,9]*, Jianhua Yang[2]*, and Jed G. Nuchtern[2,8]*

[1]Department of Ophthalmology, Shanghai Tenth People's Hospital, Tongji University School of Medicine, Shanghai 200072, P. R. China
[2]Texas Children's Cancer Center, Department of Pediatrics, Dan L. Duncan Cancer Center, Baylor College of Medicine, Houston, Texas 77030, USA
[3]Department of Pathology, University of Texas MD Anderson Cancer Center, Houston, Texas 77030, USA
[4]Department of Acupuncture, First Affiliated Hospital, Heilongjiang University of Chinese Medicine, Harbin, Heilongjiang 150040, China
[5]Department of Urology, First Affiliated Hospital, Harbin Medical University, Harbin, Heilongjiang 150001, China
[6]Department of Medical Oncology, Zhejiang Cancer Hospital, Hangzhou, Zhejiang 310022, China
[7]Xinjiang Key Laboratory of Plant Resources and Natural Products Chemistry, Xinjiang Technical Institute of Physics and Chemistry, Chinese Academy of Sciences, Urumqi, Xinjiang 830011, China
[8]Division of Pediatric Surgery, Michael E. DeBakey Department of Pediatric Surgery, Dan L. Duncan Cancer Center, Baylor College of Medicine, Houston, Texas 77030, USA
[9]Department of Stomatology, Huashan Hospital, Fudan University, Shanghai 200040, China

Running head: Wip1 inhibition in neuroblastoma

*Correspondence to:
Jed G. Nuchtern, Phone: 1-832-822-3135, Fax: 1-832-825-2141. Email: nuchtern@bcm.edu;
Jianhua Yang, Phone: 1-832-824-4572, Fax: 1-832-825-1206. Email: jianhuay@bcm.edu;
Shangfeng Liu, Tel/fax: +86-21-6562662. Email: shangfengliufudan@163.com.

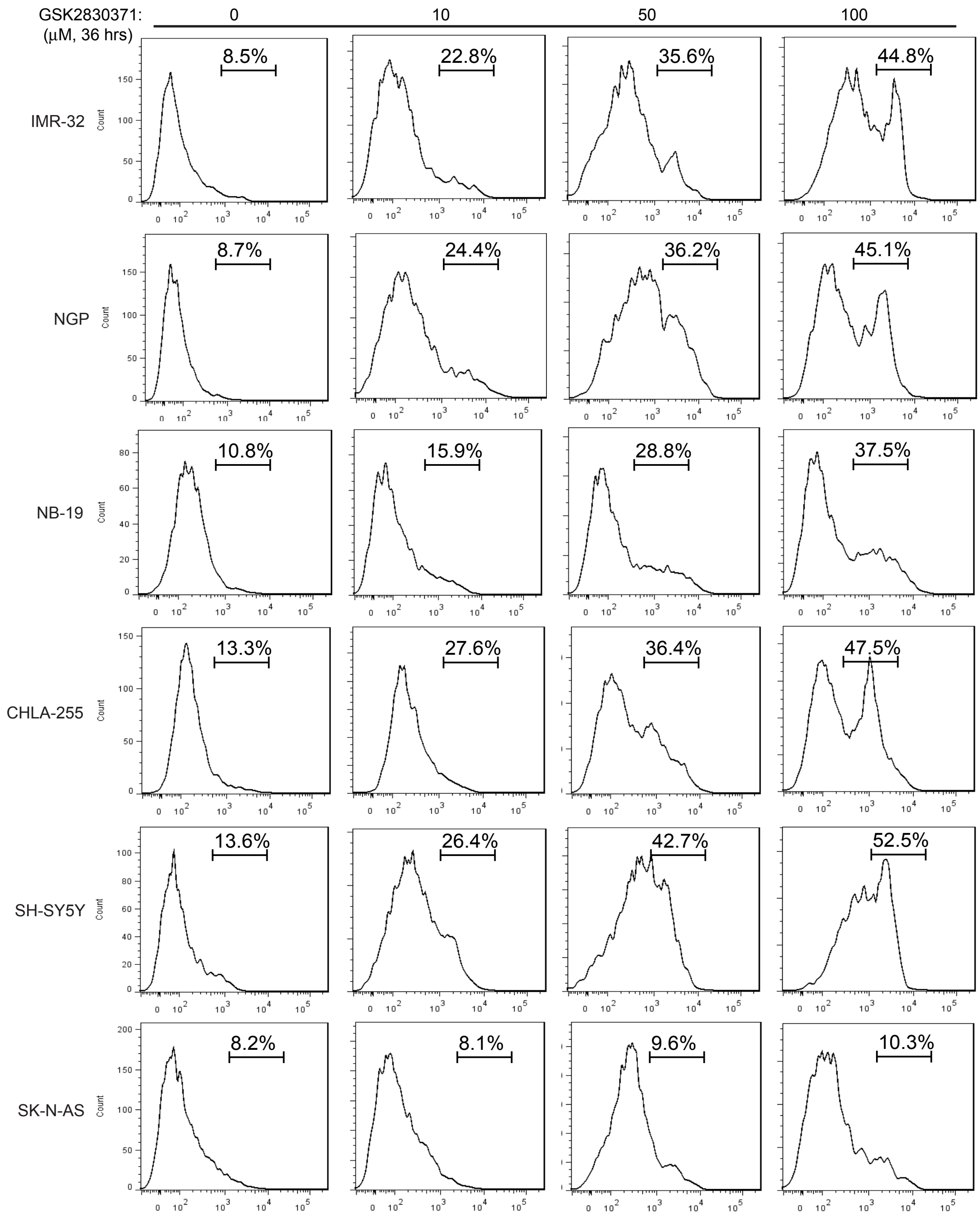

Supplemental Figure S1. GSK2830371 induces cell death in *p53* wild-type, but not in *p53* mutant NB cells. PI staining of IMR-32, NGP, NB-19, CHLA-255, SH-SY5Y, and SK-N-AS cells after treated with the indicated concentrations of GSK2830371 for 36 hrs. The cells were then analyzed by flow cytometry for the percentage of dead cells.

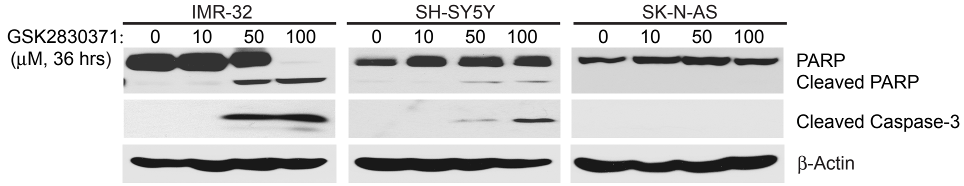

Supplemental Figure S2. GSK2830371 induces apoptosis in *p53* wild-type, but not in *p53* mutant NB cells. IMR-32, SH-SY5Y and SK-N-AS cells were treated with the indicated concentrations of GSK2830371 for 36 hrs. The cells were then lysed and subjected to SDS-PAGE followed by immunoblotting with anti-PARP and Caspase-3 antibodies. β-Actin was used as a loading control.

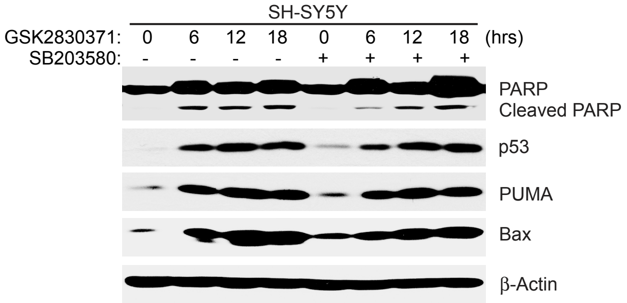

Supplemental Figure S3. p38 inhibitor SB203580 suppresses GSK2830371-induced cell death and p53 activation. SH-SY5Y cells were treated with or without p38 inhibitor SB203580 (10 µM) for 2 hrs before exposed to GSK2830371 (50 µM) for indicated time points (0-18 hrs). Cells were collected and lysed at the end of treatment followed by SDS-PAGE, and immunoblotting with the indicated antibodies. β-Actin was used as a loading control.

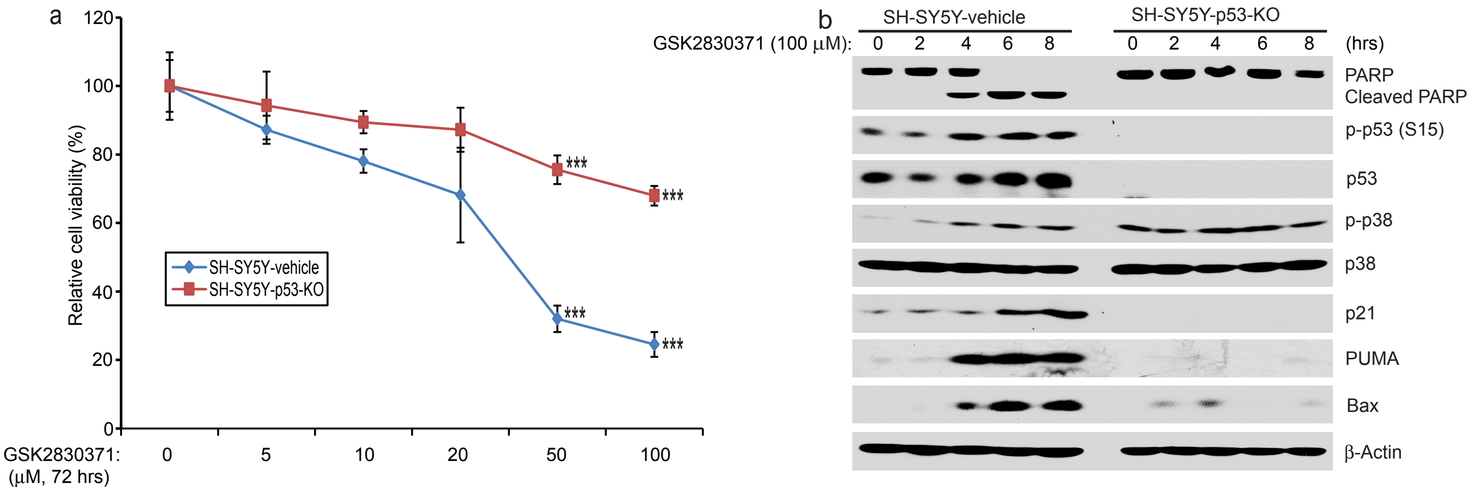

Supplemental Figure S4. p53 activation plays a major role in GSK2830371-induced cytotoxicity in SH-SY5Y cells. (**a**) SH-SY5Y-vehicle control cells and SH-SY5Y-*p53*-KO cells were seeded in 96-well plates, respectively. After incubating with the increasing concentrations of GSK2830371 for 72 hrs, cell viability was then measured by the CCK-8 assay and the results were represented as % vehicle ± S.D. P <0.001 (***) (Student's t-test, two-tailed) was indicated. (**b**) SH-SY5Y-control cells and SH-SY5Y-*p53*-KO cells were treated with GSK2830371 (100 µM) for the indicated time points (0-8 hrs). And then the cells were harvested and lysed, followed by SDS-PAGE and immunoblotting with the indicated antibodies. β-Actin was used as a loading control.

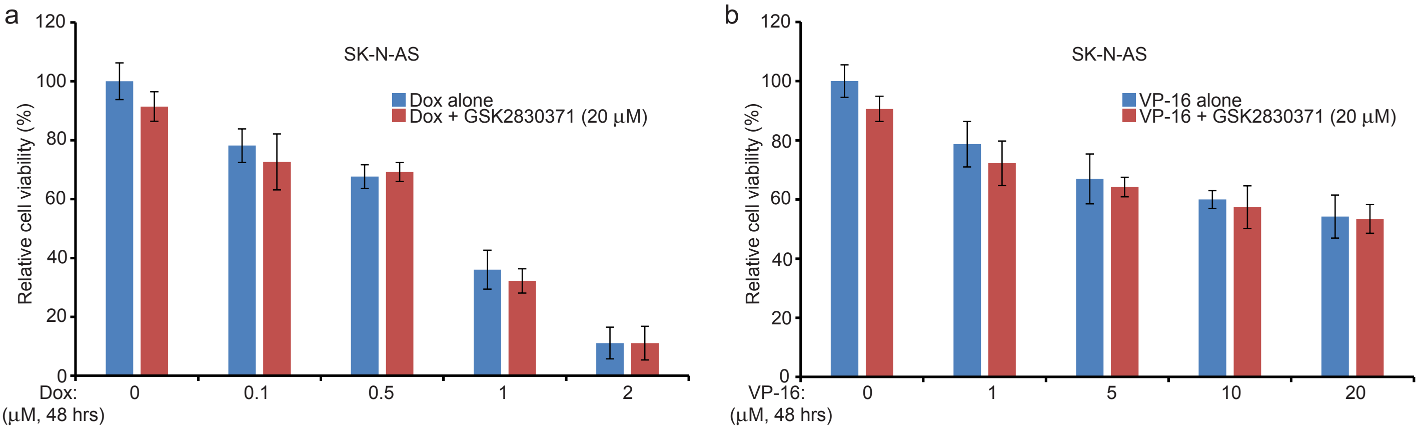

Supplemental Figure S5. GSK2830371 fails to enhance Dox- and VP-16-induced cytotoxicity in *p53* mutant SK-N-AS cells. SK-N-AS cells were seeded in 96-well plates and were incubated with the indicated concentrations of Dox (**a**) or VP-16 (**b**) plus DMSO or 20 μM of GSK2830371 for 48 hrs. Cell viability was then measured by the CCK-8 assay. Results were represented as % vehicle ± S.D.

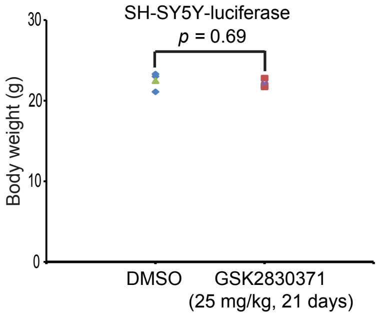

Supplemental Figure S6. GSK2830371 does not have significant effect on mouse body weights. Mouse body weights at the end of treatment (P=0.69) (Student's t-test, two-tailed).
